# Assessing Organizational Readiness for Change: Comment on "Development and Content Validation of a Transcultural Instrument to Assess Organizational Readiness for Knowledge Translation in Healthcare Organizations: The OR4KT"

**DOI:** 10.15171/ijhpm.2018.101

**Published:** 2018-10-28

**Authors:** Lisa M. Puchalski Ritchie, Sharon E. Straus

**Affiliations:** ^1^Department of Medicine, University of Toronto, Toronto, ON, Canada.; ^2^Li Ka Shing Knowledge Institute, St. Michaels Hospital, University of Toronto, Toronto, ON, Canada.; ^3^Department of Emergency Medicine, University Health Network, Toronto, ON, Canada.

**Keywords:** Organizational Readiness, Knowledge Translation, Transcultural Adaptation

## Abstract

This commentary provides an overview of the organizational readiness for change (ORC) literature over the last decade, with respect to prevailing definitions, theories, and tools to guide assessment of ORC in preparation for implementation. The development of the OR4KT by Gagnon et al is an important contribution to this body of work. This commentary highlights the strengths of the OR4KT including development based on two systematic reviews conducted by the authors to synthesize OR theory and measurement tools, and applicability to a wider range of high-income country healthcare settings through inclusion of input from a diverse group of international experts and transcultural adaptation of the tool, in the context of the literature to date. Limitations and future directions for further development of the tool are also discussed and include application of quantitative psychometric approaches and evaluation of the tool in a broader range of healthcare settings.

While organizational readiness for change (ORC) is widely recognized as necessary to implementation success,^1-5^ its assessment remains challenging. Efforts to synthesize the relevant literature over the last decade have led to a standardized definition^4^ and a conceptual framework useful in guiding operationalization^1^ of the concept. Based on their review of 106 articles, Weiner et al^4^ proposed the following definition of ORC, “the extent to which organizational members are psychologically and behaviorally prepared to implement change.”^4^ This definition has since been widely adopted in the ORC literature and provides the operational definition for much of the work in this area.^5-8^ Holt et al^1^ conceptualized OR as a framework of three dimensions, with two dimensions, structural and psychological factors, each operating at two levels, individual and organizational contexts, of the third dimension, level of analysis,^1^ and emphasized the importance of considering and assessing ORC within each of these dimensions in preparation for implementation. In addition, Weiner et al’s systematic review identified a lack of reliable and valid ORC assessment tools, leading to recent efforts to develop psychometrically sound tools for this purpose.^4^

Several ORC assessment tools, which are in the early stages of development, have recently been reported.^8-10^ Helfrich et al^9^ developed a 77-item scale based on the promoting action on research implementation in health services (PARIHS) framework^11^ and assessed scale reliability and factor structure using data from three quality improvement projects conducted in the veterans health administration (VHA), two in chronic care programs and one in an intensive care unit initiative. They found good reliability for two of three primary scales and the majority of subscales, however, as noted by the authors, further work is needed to address areas of inadequate scale/sub-scale reliability and assess criterion validity to inform refinement of the tool. Stamatakis et al^10^ developed a 23-item scale based on three theoretical frameworks and assessed the reliability of the scale with data from representatives from four chronic disease prevention programs using confirmatory factor analysis. They found good fit for two of four scales and with refinement of the remaining scales achieved good internal scale reliability and goodness of fit, although further work is needed to assess predictive validity of the tool. Shea at al^8^ developed a 15-item survey based on Weiner’s theory of ORC^2^ and assessed several aspects of its reliability through a series of four studies. These studies include vignettes regarding ORC with university students and data collected via electronic health records; and online surveys with participants from international non-governmental organizations based in the United States using data from mobile phone technology for monitoring and evaluation of international health and development programs. They found good preliminary evidence of reliability and factor structure, with further work to evaluate validity needed. While all of these scales demonstrate relatively good reliability and validity based on evaluations reported to date, all require further evaluation and validation, they represent an important step in the development of reliable and valid tools for assessment of ORC.

## Strengths of OR4KT

Gagnon et al^12^ adds to this body of work with their development of the organizational readiness for knowledge translation (KT) OR4KT tool. This work is notable for several reasons. First, in preparation for development of their ORC assessment tool, the authors conducted systematic reviews of the OR theory^7^ and of existing reliable and/or valid tools for measurement of OR in healthcare organizations.^13^ This updated synthesis of the OR literature and expert review provided the foundation for the development of the initial item pool and tool structure in terms of theoretical dimensions of relevance to assessment of ORC. Their approach ensures tool development was theory-informed and based on the best available evidence. As a result, their work expands on earlier theories including elements of Weiner’s original theory of ORC. Specifically OR4KT’s dimensions of motivation, leadership, change content, and organizational climate for change relate to Weiner’s constructs of change valence and change efficacy, and the OR4KTs dimensions of organizational support and context represent expansions of Weiner’s theory where context is noted but not explicitly included. In addition, because their systematic review focused on OR within the healthcare context, this approach may increase the tool’s applicability to such settings.

Second, unlike many of the ORC tools in development where participants and data sets were predominantly based in the United States, input in Gagnon and colleagues’ study was sought from a range of international OR and KT experts, throughout the tool development process. This broader representation of expertise and experience may help to increase utility of the final tool to a wider range of high-income countries. Third, cultural adaptation through both rigorous translation to ensure linguistic equivalence and pilot testing of face validity in two additional languages (French and Spanish) and three diverse healthcare contexts (Ontario, Quebec, and Basque region of Spain), is likely to further enhance the applicability of the final tool to a broader range of high-income country healthcare settings.

## Limitations and Future Directions

Despite the above noted strengths of the OR4KT development process, in addition to the next steps as outlined by the OR4KT authors^12^ for further reliability and validity assessment, several considerations for future assessment of the tool are worth noting. First, while the authors were able to reduce the number of items in the tool from an initial 97 to 59 and note it takes 15-20 minutes to complete, the tool remains relatively long which may limit its use among stakeholders operating in chronically overburdened healthcare organizations. Second, while the synthesis of theory and measurement tools to date, and the breadth of expert input sought during the development process of this tool are important steps in item and dimension selection. Application of quantitative psychometric approaches such as factor analysis, would be of benefit and may allow for further reduction in tool length without loss of its measurement properties. Third, similar to other works in OR measurement, the systematic reviews which informed development of the OR4KT and its initial real world validation are focused on implementation in the chronic care setting. As chronic care settings may differ substantially from their critical/emergent care counterparts, evaluation of the tool in these other clinical settings could further refine it and with appropriate adaptation as needed, extend the range of the OR4KT’s applicability. Finally, as noted by the Gagnon and colleagues applicability of OR4KT to low- and middle-income country settings is uncertain. However, given the recognized urgent need for improved implementation of evidence in low- and middle-income country healthcare settings, future work to evaluate and adapt the tool for such settings could further extend its applicability and its potential impact. Additionally, further evaluation of the tool across diverse cultural settings within high-income settings would be of interest to assess the potential impact of culture on key constructs important to assessment of ORC and tool application. Finally, while it is implied by many tool developers that ORC assessment is important to implementation planning, little detail on the specifics of potential uses of the OR4KT were noted. Future work to evaluate the potential role of ORC assessment beyond implementation planning would expand tool utility. For example, studies to assess the tool’s ability to predict sites where implementation is likely to be more effective to guide selection of initial implementation sites where large scale implementation is planned would be of interest.

## Conclusion

Based on the approach to development and initial reliability and validity assessments of the OR4KT, the tool holds promise for addressing a widely-recognized gap in the availability of tools to measure ORC. It is both hoped and believed that accurate assessment of ORC plays an essential role in implementation planning and by providing a sound measure to guide assessment, the OR4KT has potential to guide and improve implementation efforts in a variety of high-income country healthcare settings. However, further work is needed to refine the tool and to explore opportunities to extend its applicability to health care settings beyond those contributing to its development, such as critical/urgent care and low- and middle-income country health care settings.

## Acknowledgements

SES is funded by a Tier 1 Canada Research Chair in Knowledge Translation and Quality of Care and the Mary Trimmer Chair in Geriatric Medicine.

## Ethical issues

Not applicable.

## Competing interests

Authors declare that they have no competing interests.

## Authors’ contributions

Both authors conceptualized the manuscript. LMPR prepared the initial manuscript draft. SES provided critical revision of the manuscript. Both authors read and approved the final manuscript.

## Authors’ affiliations

^1^Department of Medicine, University of Toronto, Toronto, ON, Canada. ^2^Li Ka Shing Knowledge Institute, St. Michaels Hospital, University of Toronto, Toronto, ON, Canada. ^3^Department of Emergency Medicine, University Health Network, Toronto, ON, Canada
